# Editorial: Machine learning approaches for monitoring mental health and substance abuse using social media data

**DOI:** 10.3389/fpubh.2025.1590564

**Published:** 2025-04-07

**Authors:** Danila Azzolina, Erica Secchettin, Paola Berchialla, Tingshao Zhu, Aslihan Şentürk Acar, Dario Gregori

**Affiliations:** ^1^Department of Environmental and Preventive Science, University of Ferrara, Ferrara, Italy; ^2^Department of Surgery, Dentistry, Paediatrics and Gynecology, University of Verona, Verona, Italy; ^3^Statistical Unit, Department of Clinical and Biological Sciences, University of Turin, Turin, Piedmont, Italy; ^4^CAS Key Laboratory of Behavioral Science, Institute of Psychology, Chinese Academy of Sciences (CAS), Beijing, China; ^5^Department of Actuarial Sciences, Hacettepe University, Ankara, Türkiye; ^6^Unit of Biostatistics, Epidemiology and Public Health, Department of Cardiac, Thoracic, Vascular Sciences and Public Health, University of Padua, Padua, Veneto, Italy

**Keywords:** social media, artificial intelligence, mental health, substance abuse, machine learning, text mining

The rapid growth of social media platforms has changed how individuals express and share personal habits and experiences, including those related to mental health and substance use (1). In parallel, advances in machine learning (ML) have provided powerful tools for analyzing this rich and abundant data source, creating new possibilities for public health research (2).

This Research Topic brings together several studies that explore the intersection of these two domains, employing ML techniques to monitor, analyze, and predict mental health conditions and substance abuse patterns using social media data, illustrating how innovative approaches could impact public health monitoring through the lens of social media data.

## Mental health perspective

The mental health-focused articles in this collection emphasize how machine learning and social media data can be harnessed to monitor public attitudes, predict negative mental health outcomes, and explore the potential of AI-driven tools in improving access to mental health care, offering new perspectives on early intervention and support strategies. In this framework, Li tackles the prediction of negative attitudes toward suicide on the Chinese social media platform Sina Weibo. Combining psycholinguistic features with machine learning models, the study provides a framework for identifying public attitudes toward suicide. Unlike previous research that focused primarily on stigmatizing language, this study covers a broader range of negative attitudes, such as dismissive and indifferent tones, offering a more in-depth understanding of public discourse. The model's high predictive accuracy, combined with its rigorous external validation, underlines the importance of incorporating diverse forms of negative attitudes into predictive frameworks for mental health monitoring (Li). This approach is important for reducing stigma and promoting informed, compassionate conversations around the mental health.

Integration of artificial intelligence into mental health care is further illustrated by Grosshans et al., who present a case study on the use of conversation-based AI (CAI) for managing anxiety disorders. The patient in this study initially uses a chatbot for self-management of social anxiety, gradually transitioning to human-based psychiatric care. This case highlights how technology can lower the threshold for seeking traditional medical assistance, particularly for individuals who might otherwise avoid professional help due to the stigma or discomfort. The study also emphasizes the importance of blending AI-driven support with human expertise to ensure safety and effectiveness in care (Grosshans et al.). While the chatbot provides valuable psychoeducation and therapeutic suggestions, the human intervention helps to validate these strategies and offers ongoing guidance, creating a complementary care model.

## Substance use perspective

The articles on substance use highlight the power of machine learning to analyze social media discussions, providing real-time insights into public perceptions, regional patterns, and emerging trends related to tobacco consumption and drug safety, ultimately supporting more targeted public health interventions.

An important theme emerging from these studies is the use of natural language processing (NLP) to analyze unstructured text data from social media. For example, Dong et al. developed a BERT-based language model to extract drug-related adverse events from social media, addressing the limitations of traditional pharmacovigilance systems, such as the FDA's Adverse Event Reporting System (FAERS). Their findings highlight the potential of social media to complement existing surveillance systems by offering immediate insights into adverse drug reactions. The model achieves high F1 scores in detecting adverse event mentions across multiple datasets, demonstrating its robustness and practical utility in pharmacovigilance practices (Dong et al.). This approach not only facilitates real-time monitoring capabilities but also bridges the temporal gap inherent in conventional reporting systems, offering a more proactive response to emerging safety issues.

Similarly, Castillo-Toledo et al. explore the content of tobacco-related tweets, providing regional insights into public perceptions and user opinions. Their study analyzes more than 56,000 tweets in English and Spanish, revealing that healthcare professionals are the most frequent contributors to the discussion, often promoting accurate and evidence-based health information. The geolocation data revealed regional disparities, with Africa showing the lowest engagement in discussions about the health risks of tobacco. This finding aligns with the continent's relatively underdeveloped anti-tobacco policies, emphasizing the importance of geolocational analyses for identifying public health gaps and tailoring interventions accordingly (Castillo-Toledo et al.). This work demonstrates how social media can serve as a mirror of public health awareness and policy impact across different regions.

Together, these studies reflect the potential of machine learning applications to social media mining to facilitate public health monitoring and intervention strategies. Using social media data, researchers can gain real-time insights into public health trends, track evolving behaviors, and detect emerging risks.

## Challenges and future directions

The use of social media data comes with its own set of challenges. Data quality and representativeness remain significant issues, as social media content is often noisy and culturally biased. This can affect the accuracy and generalizability of machine learning models (3). Furthermore, ethical considerations around privacy, informed consent, and the potential misuse of predictive models must be carefully addressed to ensure responsible and fair applications (4).

Future research should prioritize the development of adaptive models capable of handling the dynamic nature of social media content while maintaining transparency and fairness in predictions. Collaborative efforts between data scientists, public health professionals, and policymakers are important for translating these technological advances into actionable strategies that can improve health outcomes. Moreover, integrating machine learning insights into existing health infrastructures will require the development of clear ethical guidelines and standardized protocols.

In conclusion, the contributions to this Research Topic offer a snapshot of how machine learning reshapes public health surveillance through social media. From monitoring adverse drug reactions to analyze public attitudes toward suicide and integrating AI into mental health care, these studies highlight the transformative potential of combining computational methods with digital data sources. We hope that this collection inspires further interdisciplinary collaboration and innovation, ultimately contributing to more effective, data-driven public health solutions.
